# CRISPRessoSea: streamlined analysis and comparison of pooled amplicon CRISPR screens

**DOI:** 10.1186/s12859-026-06518-9

**Published:** 2026-06-13

**Authors:** Samuel Coleman, Jacob Tye, Daxton Furniss, Robert Sainsbury, Abhay Rastogi, Xincen Xi, Balazs Murnyak, Jason Bell, Joseph Skeate, Minjing Wang, Beau Webber, Branden Moriarity, Kendell Clement

**Affiliations:** 1 Department of Biomedical Informatics, University of Utah, Salt Lake City, UT USA; 2 Department of Pediatrics, Masonic Cancer Center, Center for Genome Engineering, University of Minnesota, Minneapolis, MN USA

**Keywords:** CRISPR, Genome editing, Off-targets, Pooled amplicon sequencing analysis

## Abstract

**Background:**

CRISPR genome editing enables precise modification of genomic targets but may also induce unintended edits at off-target sites with similar sequences. Pooled amplicon sequencing can assess on- and off-target editing across many samples, yet analyzing, aggregating, and visualizing results from multiple pooled experiments remains challenging. Tools to simplify and standardize these analyses are needed to provide reproducible and comparable interpretation of editing data.

**Results:**

We developed CRISPRessoSea, a software package that processes, compares, and visualizes genome editing rates from pooled amplicon sequencing experiments. The tool provides standardized workflows for analyzing editing across multiple targets and samples, supports both nuclease- and base-editing modalities, and generates clear, data-rich summaries suitable for downstream interpretation.

**Conclusions:**

CRISPRessoSea facilitates reproducible, scalable analysis of CRISPR editing outcomes across diverse experimental designs, enabling more efficient and transparent assessment of genome editing specificity. The software is freely available at https://github.com/clementlab/CRISPRessoSea.

**Supplementary Information:**

The online version contains supplementary material available at https://doi.org/10.1186/s12859-026-06518-9.

## Background

CRISPR editing is directed to a specific genome target by the nucleotide sequence encoded in the guide RNA (gRNA) [1]. Different genomic targets can be specified by altering the gRNA sequence [2]. This flexibility has contributed to the successful application of CRISPR genome editing technologies to many research and clinical applications [3]. However, off-target editing activity may occur at sites that have sequence similarity to the gRNA [4, 5], at a rate that is not easily predicted using computational methods [6–9]. Potential off-target sites can be nominated using computational [10–12] or molecular-based assays [13–17], but editing at nominated sites must be validated experimentally to confirm editing of DNA sequences. CRISPR editing at on- and off-targets can be profiled using pooled amplicon sequencing experiments where PCR primers targeting multiple sites are pooled and used to amplify multiple genomic targets in bulk samples in the same PCR reaction. A sequencing library containing amplified fragments from all genomic sites is sequenced to yield DNA sequences for each site. Sequencing is normally performed using next-generation sequencing, which enables high-throughput and high-resolution profiling of sequences at potential off-target editing sites. In order to analyze sequenced reads for a specific sample or condition, reads must first be demultiplexed or assigned to the specific genomic target. Next, all reads assigned to a single genomic target are analyzed for the presence of genome sequencing edits by comparing read sequences to a reference sequence. Finally, genome editing rates can be aggregated across all sites and reported to the user.

Many computational tools have been developed to analyze CRISPR genome editing outcomes from next-generation sequencing data. CrispRVariants [18] quantifies edits and allows for visual comparison of variant alleles at a single target between samples. CRIS.py [19] allows comparison of the sequence length of alleles at a single target between samples. CRISPResso2 [20] run in CRISPRessoBatch mode can be used to visualize edits across samples, including specialized visualizations for cleaving nucleases, which produce indels, as well as for base editors, which create programmed substitutions from one base to another. Several software packages enable comparison of edits across multiple locations between two samples. AmpliCan [21] identifies edits by comparing sequences in a treated sample to a mock sample. CRISPECTOR [22] performs this comparative analysis and adds quantification of translocations between target locations. CRISPECTOR2 [23] enables allele-specific quantification of indels. CRISPResso [24] or CRISPResso2 [20] run in CRISPRessoPooled mode can be used to analyze multiple targets in a single sample, while CRISPRessoPooledCompare mode can aggregate editing rates across multiple samples (See comparison to CRISPRessoPooled in Supplementary Note 2).

However, while these software tools can analyze one or two pooled experiments, comparing more than two pooled experiments requires custom processing and statistical test development. This creates a barrier to the interpretation of pooled analyses, making it difficult for the community to analyze and evaluate results without standardized tools and methods. Meanwhile, pooled amplicon sequencing assays to profile multiple targets are becoming more accessible due to the ease in computational or biochemical identification of potential off-target sites and the decreased cost associated with sequencing. At the same time, demand for exhaustive off-target editing quantification is driven by the necessity to evaluate off-target editing in clinically-focused genome editing applications [25–27].

**Fig. 1:**
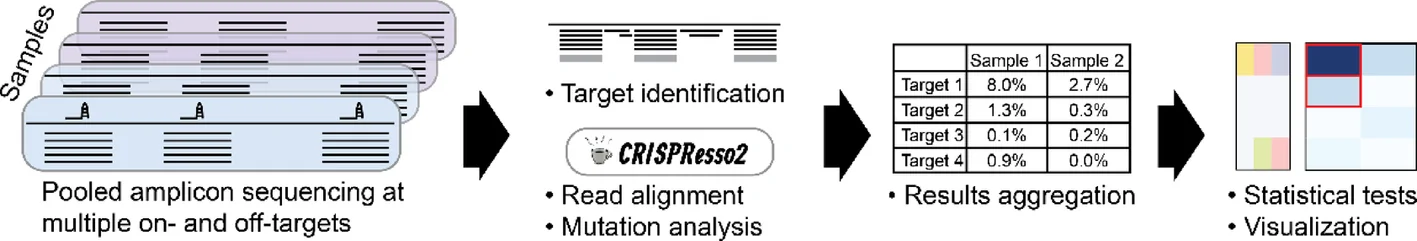
Overview of CRISPRessoSea functionality and outputs. Schematic overview of CRISPRessoSea processing. Pooled amplicon sequencing experiments profile editing at multiple genomic targets across multiple samples or conditions. CRISPRessoSea optionally performs target identification by identifying enrichment of read alignments corresponding to amplified loci. For each sample, sequencing reads are processed with CRISPResso2 to align reads and quantify editing outcomes. Editing metrics are then aggregated across targets and samples. Finally, CRISPRessoSea performs statistical comparisons between experimental conditions and generates a comprehensive interactive report summarizing editing efficiencies, mutation profiles, and statistical test results

To address this need, we have developed CRISPRessoSea, which performs CRISPR genome editing analysis across many samples of pooled sequencing experiments, capable of characterizing editing at on- and off-targets across experimental conditions. The program accepts raw sequencing files as input, performs an analysis at each site in each sample, performs a statistical analysis of editing, and produces plots and reports detailing editing rates (Fig. 1, See implementation details in Supplementary Note 1).

## Implementation

### Processing

CRISPRessoSea is designed to efficiently process pooled genome editing data across many targets and experimental conditions. Each sample is analyzed independently using its corresponding FASTQ file, which contains sequencing reads from multiple on- and off-target sites. CRISPRessoSea is based on the popular CRISPResso [20] package for genome editing analysis. Initially, each sample is processed independently based on its respective FASTQ file containing sequencing reads from multiple targets. CRISPRessoSea uses CRISPRessoPooled to demultiplex reads to the correct genomic target based on sequence alignment. After assignment to a genomic target, reads are re-aligned to the target reference sequence using a biologically informed alignment algorithm that prioritizes alignments where insertions and deletions are assigned to the CRISPR cleavage site [20]. Next, insertions, deletions, and substitutions are quantified within a quantification window set by the user. The quantification window is designed to remove the effects of sequencing errors or other sources of sequence variation away from the CRISPR cut site. Finally, CRISPRessoSea aggregates editing rates across samples and genomic targets, creating summary data files, informative figures, and an intuitive summary report.

### Results aggregation and visualization

CRISPRessoSea aims to balance the presentation of granular editing data (such as the frequencies of specific nucleotide sequences at targets), with the need for a concise, easily interpretable summary of editing outcomes. To achieve this, the software supports two complementary aggregation methods. The first aggregates the percent of reads containing any edit (insertion, deletion, or substitution) at the predicted guide cut site for each sample, providing an overall measure of editing prevalence. The second reports the maximum editing rate at the most frequently modified base within each target, a strategy used previously to capture the peak activity of an editor [25]. To accommodate the analysis of a variety of CRISPR nucleases and designs, we have implemented maximum-editing rate aggregation methods for cleaving nucleases as well as for base editors. For nucleases that introduce insertions or deletions (indels), we define the editing rate for a target as the indel frequency at the nucleotide position with the highest observed indel frequency. For base editors, we report the highest frequency of the intended substitution within the quantification window: C → T for cytosine base editors and A → G for adenine base editors. This approach provides users with a clear summary of editing activity across samples and target sites, enabling rapid identification of the most active or variable sites. By highlighting sites with the highest editing rates, these summaries help prioritize which samples and targets may warrant closer inspection.

To provide a comprehensive graphical overview of genome editing outcomes, CRISPRessoSea generates several plots that visualize the aggregated editing data. The main plot displays the genomic sequence of each target, highlighting sequence differences relative to the reference guide (typically the on-target site). Summarized editing rates are shown as a heatmap, with each row representing a target and each column representing a sample. An additional bar plot aggregates editing rates by target, and if sample groups are defined, editing rates are visualized by group to allow for comparisons between groups. These plots are presented in an interactive HTML report, which includes explanatory legends and links to the raw data used to generate each figure. Additionally, the report provides direct links to the associated CRISPRessoPooled analyses, enabling users to explore the precise alleles detected at each target in each sample. Together, this report enables high-level visual interpretation of editing activity while also offering the ability to drill down into detailed, allele-level data.

### Tests for editing activity

Statistical testing of genome editing rates is essential for the objective identification of true editing events, particularly when evaluating low-frequency modifications that might otherwise be mistaken for background noise. These methods are especially critical in the analysis of potential off-target activity, which typically occurs at lower rates than on-target editing but can have important implications in both research and clinical settings. Several sources of noise—such as sequencing errors, PCR artifacts, and endogenous sequence variation (e.g., SNPs)—can artificially inflate measured editing rates, even in untreated control samples. To account for this, some approaches apply simple subtraction of editing rates in control samples from those in treated samples to remove background signal [16]. Alternatively, statistical tests can be used to assess whether observed differences are significant while accounting for factors such as sequencing depth. For instance, Fisher’s exact test has been applied to evaluate whether the proportion of edited reads in a treated sample is significantly higher than in a control sample, while incorporating uncertainty due to coverage [28]. When multiple replicates or samples are available, more robust statistical tests such as the Wilcoxon rank sum test [29, 30] or Student’s t-test [29, 31] can be used to assess differences in editing rates between experimental groups. Differences in sequencing read depth can be modeled using negative binomial regression [25]. However, the choice of test is often ad hoc and inconsistent across studies. To address this gap, we provide a flexible framework that supports multiple statistical approaches for detecting significant editing events in single samples or across groups or conditions.

We have implemented five methods for assessing editing rate significance, ranging from threshold-based filters to formal statistical tests: (1) Hard Cutoff: This test simply checks whether the editing rate for a single target exceeds a user-specified cutoff. If it does, the target is marked as significant. (2) Mean Difference: For each target, the mean editing rate for two user-specified groups is calculated. If the difference in means exceeds a user-specified cutoff, the target is marked as significant. (3) Mann–Whitney U test: For each target, the Mann–Whitney U test is used to determine whether there is a significant difference between the distributions of the two user-specified groups. *P*-values are corrected for multiple testing using the Benjamini–Hochberg procedure. (4) T-test: For each target, a Welch’s t-test (unequal variance t-test) is performed to compare editing rates between two groups. *P*-values are corrected for multiple testing using the Benjamini–Hochberg procedure. (5) Negative Binomial Test: For each target, a negative binomial regression is performed comparing editing rates between two groups. The model accounts for the total number of reads (as an offset) and tests whether group assignment is a significant predictor of editing frequency. *P*-values are corrected for multiple testing using the Benjamini–Hochberg procedure.

Different statistical approaches may be appropriate depending on experimental design and sequencing characteristics. Hard cutoff and mean-difference methods provide simple and interpretable summaries but may be sensitive to background sequencing noise. Method 1 (hard cutoff) can be applied to studies where no replicate groups are defined. The other methods require at least two replicates per user-specified group. Welch’s t-test may perform well when replicate numbers are sufficient and editing frequencies are approximately continuous and normally distributed, while the Mann–Whitney U test provides a nonparametric alternative that may be more robust to outliers but less sensitive at small sample sizes. Negative binomial regression explicitly models sequencing count data and differences in sequencing depth and may therefore provide improved sensitivity for low-frequency editing events, particularly in experiments with variable coverage. To evaluate the behavior of these statistical approaches across a range of editing efficiencies, sequencing noise levels, and replicate numbers, we performed simulation studies using simulated pooled amplicon sequencing datasets. Analyses presented in Supplementary Note 4 show tradeoffs between sensitivity and specificity across replicate numbers, editing rates, and background noise conditions. By providing accessible methods to assess editing significance, we aim to help the genome editing community better interpret reported editing differences and establish clearer standards for determining which off-target sites may warrant further investigation.

## Results

We used CRISPRessoSea to reanalyze pooled amplicon sequencing data across different experimental conditions, demonstrating its ability to process and summarize editing outcomes from both nuclease-based editors that induce insertions and deletions and base editors that mediate targeted nucleotide conversions. In both cases, CRISPRessoSea provides standardized and reproducible processing from raw sequencing files to data-rich summaries with clear visual outputs, facilitating interpretation and supporting quantitative analysis of genome editing experiments.

### CRISPRessoSea identifies on- and off-target editing by CRISPR/Cas9

**Fig. 2:**
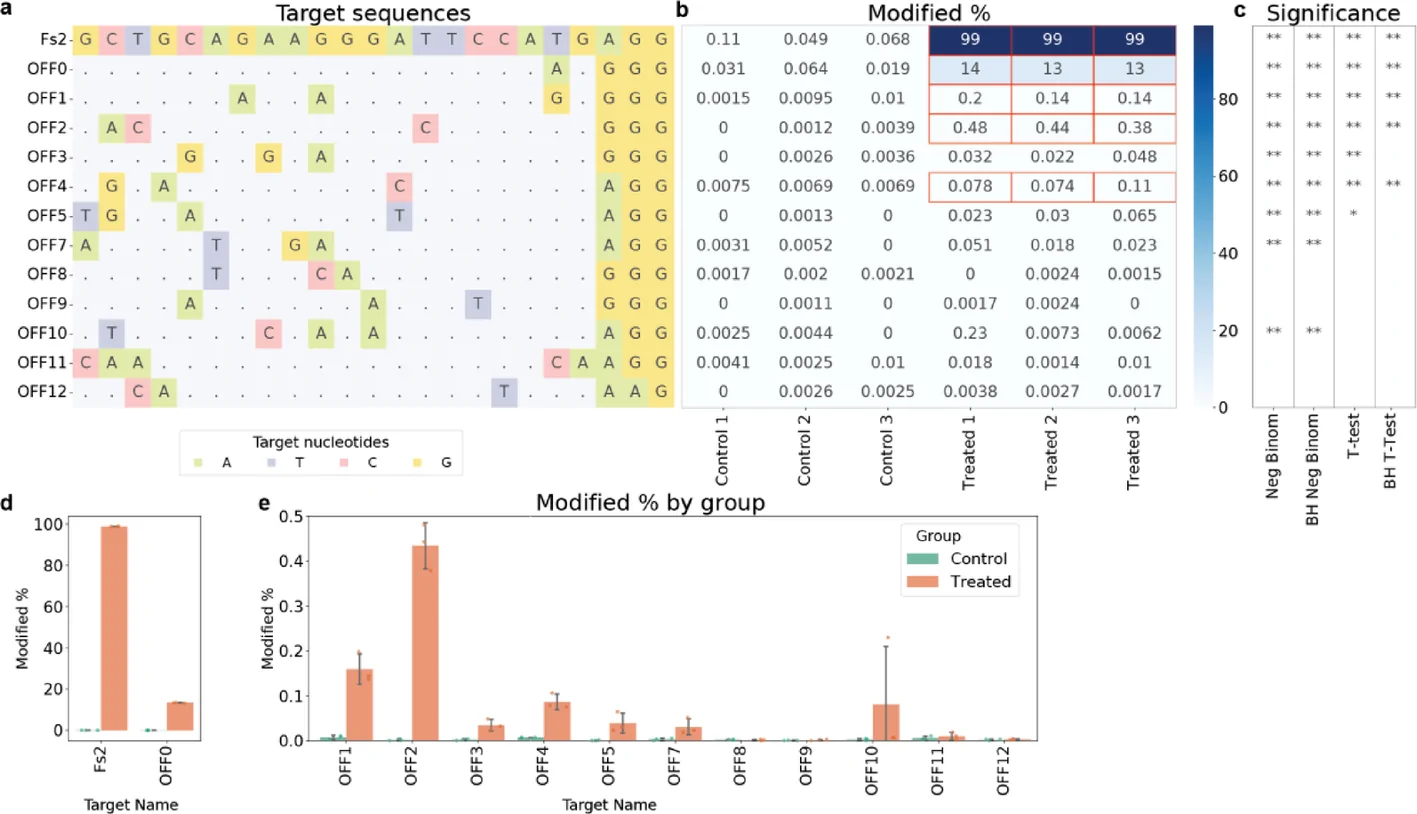
Analysis of Cas9 editing using CRISPRessoSea. CRISPRessoSea was used to quantify editing at the FANCF site-2 on-target locus and 12 DISCOVER-Seq+ nominated off-targets. **a** Nucleotide plot showing the on-target sequence (top) and off-target sequences. Matches to the on-target are shown as gray dashes; mismatches are labeled as colored bases. **b** Heatmap of modification rates for each target. Sites with significant editing (Benjamini–Hochberg–corrected Welch’s t-test, *p* < 0.01) are outlined in red.** c** Significance of editing at each site based on statistical tests (*p < 0.05; **p < 0.01). **d**–**e** Bar plots showing group-wise editing rates at on- and off-target sites for control and Cas9-treated samples

Nomination of CRISPR–Cas9 off-targets can be performed using computational prediction or biochemical assays that map double-strand breaks (DSBs) genome-wide [32]. DISCOVER-Seq+ is one such biochemical assay. For a guide targeting *FANCF* site 2 in K562 cells, DISCOVER-Seq+ was used to nominate candidate off-target sites, which were then measured by amplicon sequencing [31]. CRISPRessoSea analysis revealed high on-target editing in Cas9-treated samples (mean 91.1%) compared to untreated controls (0.07%), along with lower but detectable editing at several off-target sites. Visualization outputs highlight expected trends, including higher editing at an off-target with a single mismatch (OFF0) relative to a site with three mismatches (OFF1) (Fig. 2). Exact commands, guide files, and sample files used to generate these analyses are provided in Supplementary Note 3.

We next applied CRISPRessoSea’s statistical testing methods to evaluate the significance of editing at on- and off-target sites. Because all sites were nominated by DISCOVER-Seq+ , editing could plausibly occur at any of them, although detection may be limited by constraints of amplicon sequencing (e.g., large deletions eliminating primer-binding regions). To assess how different statistical approaches perform under these conditions, we evaluated editing rates at each site using multiple tests. Using the negative binomial test, nine sites were significant at *p* < 0.01, and all remained significant after Benjamini–Hochberg correction. Welch’s t-test identified six sites significant at *p* < 0.01 and one additional site at *p* < 0.05; after correction, five sites remained significant at *p* < 0.01. Although implemented in CRISPRessoSea, Mann–Whitney U tests were not performed because the SciPy implementation does not reliably handle groups with fewer than four replicates. These results indicate that the negative binomial approach is the most sensitive test for this dataset and illustrate that stringent multiple-testing correction may be overly conservative when all tested sites are likely to be true editing candidates due to nomination by biochemical assays.

### CRISPRessoSea quantifies on- and off-target editing by base editors

Natural killer (NK) cells’ unique ability to kill transformed cells expressing stress ligands or lacking major histocompatibility complexes (MHC) has prompted their development for immunotherapy. However, NK cells have shown only moderate responses against cancer in clinical trials. Highly efficient single and multiplex base editing can be achieved with concurrent transposon-driven insertion of a CAR transgene, resulting in significantly enhanced NK cell function in vitro and in vivo with minimal off-target base edits during multiplex engineering [33].

**Fig. 3:**
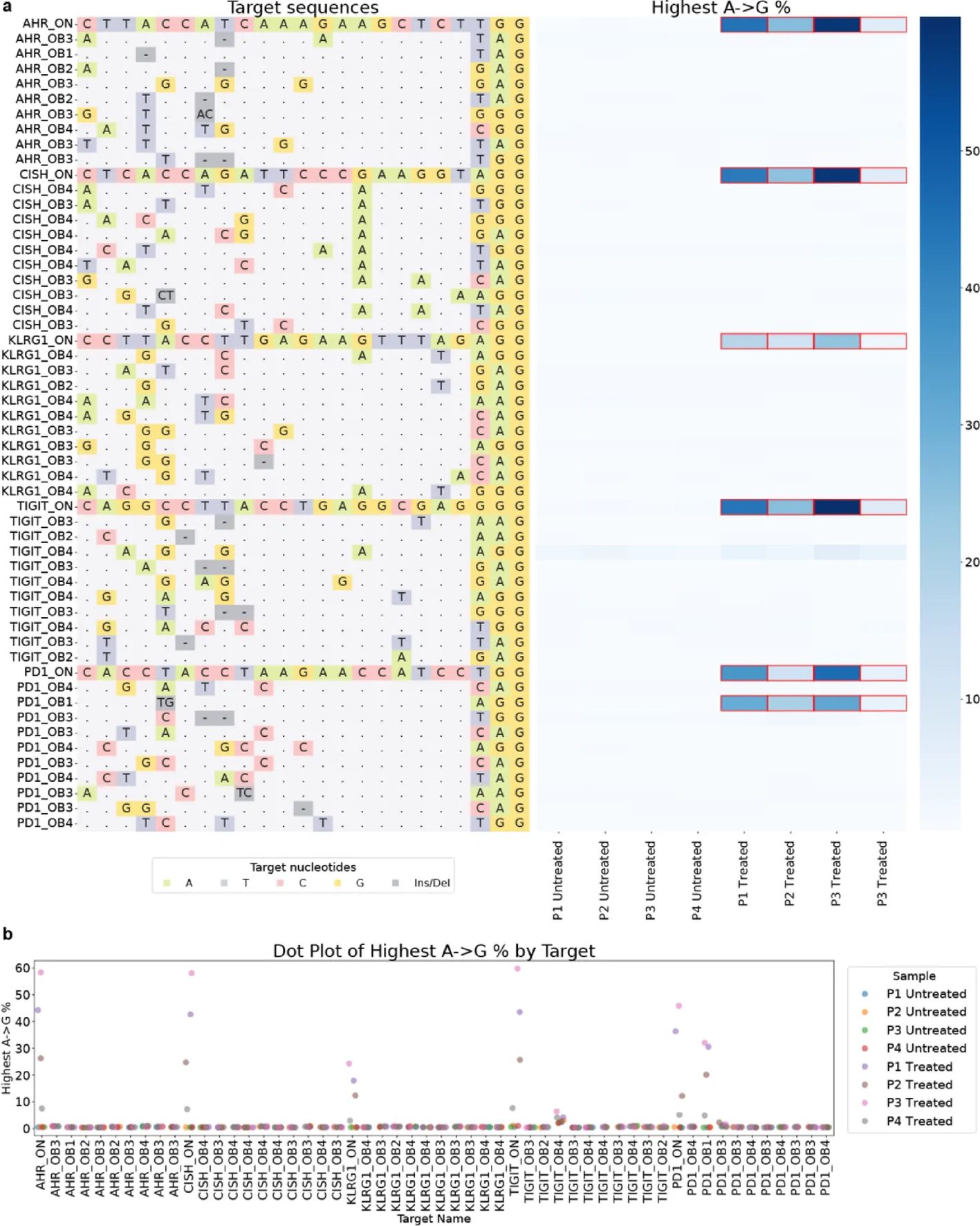
Analysis of A > G base editing using CRISPRessoSea. CRISPRessoSea was used to analyze multiplexed A > G base editing across five targets and their associated off-targets in four patient-derived cell lines, comparing editing rates between control and treated samples. **a** Nucleotide plot showing the sequences of on- and off-target sites (left) and corresponding A > G editing rates (right). Sites with significant editing (*p* < 0.01, Benjamini–Hochberg–corrected negative binomial test) are highlighted in red. **b** Dot plot displaying editing rates colored by sample

Pooled amplicon sequencing was used to profile editing activity at on- and off-targets for multiplex editing by gRNAs targeting *AHR*, *CISH*, *KLRG1*, *TIGIT*, and *PDCD1* in NK cells isolated from four healthy donors. Because base editing may affect multiple bases within the editing window, CRISPRessoSea measures the highest A- > G editing rates within the editing window, defined as a 20bp region centered 15 bp upstream of the PAM end of the guide. Although editing efficiencies varied among donors, all targets were successfully edited in all samples, and no editing was detected in unedited controls (Fig. 3). Editing rates were statistically compared between edited and unedited populations using the negative binomial method as implemented in CRISPRessoSea. Base editing was significant at all on-target sites (*AHR*: 1.8 × 10⁻⁷; *CISH*: 1.8 × 10⁻⁷; *KLRG1*: 2.0 × 10⁻5; *TIGIT*: 2.0 × 10⁻⁶; *PDCD1*: 2.1 × 10⁻⁶; BH-corrected). Off-target editing was not significant except for one *PDCD1* off-target located in a non-coding region previously reported (1.1e-06, BH-corrected negative binomial regression).

These results demonstrate CRISPRessoSea’s ability to rapidly analyze and statistically quantify editing outcomes from base editors across multiple targets. Its integrated visualizations further enable straightforward inspection of guide sequences and nearby bases at potential off-target sites, facilitating transparent and reproducible assessment of editing specificity.

## Discussion

CRISPRessoSea provides a scalable and flexible framework for processing, aggregating, visualizing, and evaluating genome editing data across hundreds of genomic targets and samples. It builds on the biologically-aware alignment strategy of the CRISPResso [20] suite to generate high-quality alignments, reducing the need for manual inspection of individual sites. The tool supports aggregation for cleavage-based genome editors and base editors, enabling analysis across editing platforms. CRISPRessoSea allows users to transition between high-level editing summaries and detailed, nucleotide-level modification profiles. This combination makes it well-suited for both exploratory analyses and the generation of publication-ready figures.

Choosing the appropriate statistical test to identify potential editing sites, particularly off-targets, remains a challenge in the field of genome editing. While CRISPRessoSea currently supports basic comparisons between two groups, more complex study designs may require additional statistical approaches. In some cases, standard multiple-hypothesis *p*-value correction may be overly conservative, especially when off-target activity is biologically plausible at all sites included in the pooled sequencing panel. To support more nuanced analysis, we provide raw summary data that users can apply in their own analyses, including comparisons across multiple groups, time points, or mixed-effects models. Additionally, formal benchmarking of statistical approaches across differences in replicate number, sequencing depth, and biological variability will be important for establishing best practices and improving the reliability of editing detection across studies.

Future development could expand CRISPRessoSea’s ability to quantify editing in the context of genomic variation and support additional genome editing technologies. For example, non-reference variants can inflate apparent editing rates. With our current analysis, this inflation should affect treated and control samples equally and not lead to significance, but support for phased or multi-allelic alignments could improve accuracy, particularly in clinical studies. Additional improvements could support analysis of technologies like prime editing, where the types and frequencies of off-target edits are less well understood. While users can currently use the indel report to monitor cleavage activity, future versions could include dedicated modules to summarize prime editing outcomes, especially for non-canonical edits.

## Conclusions

CRISPRessoSea is part of a growing ecosystem of tools designed to make genome editing analysis accessible to a broader range of researchers. This tool combines streamlined processing with intuitive visualizations and robust statistical and annotation capabilities, empowering users to explore their data with confidence, identify biologically meaningful effects, and communicate findings with clarity. We anticipate that this tool will expand access to high-quality genome editing analysis and support the application of genome editing technologies across biology and medicine.

## Availability and requirements

Project name: CRISPRessoSea

Project home page: https://github.com/clementlab/CRISPRessoSea

Operating system(s): Linux

Programming language: Python

Other requirements: None

License: MIT

Any restrictions to use by non-academics: Requires a CRISPResso2 license for commercial use.

## Supplementary Information

Below is the link to the electronic supplementary material.

Supplementary Material 1.

## Data Availability

Analyzed datasets are available from the authors of the corresponding publications [31, 33]. Amplicon sequencing data for NK cells is available at PRJNA1418185.

## Abbreviations

CRISPR: Clustered regularly interspaced short palindromic repeats
gRNA: Guide RNA
PCR: Polymerase chain reaction
DSB: Double-strand break
NK: Natural killer (cells)
MHC: Major histocompatibility complex
BH: Benjamini–Hochberg (correction)
FASTQ: File format for sequencing reads
