# Supplementary material for: CRISPRessoSea: streamlined analysis and comparison of pooled amplicon CRISPR screens
Source: BMC Bioinformatics. 2026 Jun 13;27:210. doi: 10.1186/s12859-026-06518-9 (PMC13543416; doi:10.1186/s12859-026-06518-9)
Supplement: Supplementary file 1 [file 12859_2026_6518_MOESM1_ESM.docx]

# Supplementary Note 1: CRISPRessoSea implementation details

CRISPRessoSea operates in three primary running modes to streamline the preparation, processing, and replotting of large-scale pooled sequencing experiments to measure and compare CRISPR genome editing.

### MakeGuideFile Mode

This mode enables users to generate a comprehensive target file that includes both on- and off-target sites for one or more guide RNA sequences. Off-target sites are computationally predicted using Cas-OFFinder^1^, which reports genomic coordinates, sequences, and mismatch counts for each potential target. The MakeGuideFile functionality is especially useful for designing pooled experiments to profile editing activity at predicted off-target sites, or for preparing analyses when the off-target sequences or locations are not already known. The output of this mode is a target file compatible with CRISPRessoSea’s *Process* mode.

Inputs:

- --guide_seq (required): Comma-separated guide RNA sequences. These are the sequences for which off-target
- --guide_name: Comma-separated guide names (optional). Each guide will be annotated by the respective guide_name.
- --pam: PAM sequence (default: NGG). This PAM sequence is used by Cas-offinder in identifying off-targets.
- --genome_file (required): Reference genome FASTA file used by Cas-offinder to identify off-targets.
- --max_mismatches: Maximum allowed mismatches between guide sequences and off-targets (default: 4).
- --max_dna_bulges, --max_rna_bulges: Maximum allowed bulges in off-target nomination (Cas-OFFinder 3 required).
- --output_folder: Output directory.
- --file_prefix: Prefix for output files.

Outputs:

- [file_prefix].guide_info.txt: Table of all predicted on- and off-targets for the guides, including sequence, PAM, mismatch/bulge info, and genomic location.
- Cas-OFFinder input/output/log files for reproducibility.

### Process Mode

This mode processes a set of pooled CRISPR/Cas sequencing datasets, assigning targets to regions and generating summary statistics and plots. The pipeline begins by aligning sequencing reads to the reference genome using Bowtie2.^2^  Aligned reads are passed to CRISPRessoPooled^3^ which identifies regions with high read coverage. Targets (e.g., on- or off-targets) are matched to these regions based on their genomic coordinates, and reference sequences for each amplicon are extracted accordingly. Each sample is then analyzed using CRISPRessoPooled, applying a quantification window centered on the guide position within the amplicon. Edits—such as indels and base substitutions—within this window are used to compute editing rates, while variation outside the window is excluded to minimize the impact of sequencing noise. CRISPRessoSea then aggregates results across all samples. For each target, the site within the quantification window with the highest editing rate is reported. Final outputs include summary data tables and an interactive HTML report for exploring editing outcomes across samples and targets.

Inputs:

- --sample_file (required): Tab- or Excel-formatted file listing samples, with columns: Name, fastq_r1, fastq_r2 (optional), and optionally group.
- --target_file (required): Tab- or Excel-formatted file listing targets, with columns: Guide, Sequence, PAM, #MM, Locus. This file can be generated using the MakeGuideFile mode, or input from amplicon sequencing design tools.
- --genome_file (required): Path to the reference genome FASTA file (Bowtie2-indexed).
- --output_folder: Output directory for results (default: auto-named).
- --gene_annotations: Optional gene annotation file (BED or similar).
- Various parameters for CRISPResso2 and analysis (see help for all options).

Outputs:

- aggregated_stats_all.txt: Table of editing rates and summary statistics for all targets and samples.
- Plots (heatmaps, dot plots) in PDF/PNG format.
- CRISPRessoSea_info.json: Run metadata and summary.
- HTML report summarizing results.
- Intermediate files: region assignments, target-region matches, per-sample summaries.

### Replot Mode

This mode enables users to regenerate and customize plots from a previously completed analysis without reprocessing the underlying sequencing data. Users can reorder samples or targets, switch between statistical tests, or adjust visual properties such as figure size and color palette. This allows for efficient iteration on figure layout and formatting, particularly valuable in large experiments where computational resources may be a limiting factor.

Inputs:

- --reordered_stats_file (required): Aggregated stats file (e.g., aggregated_stats_all.txt), possibly reordered.
- --reordered_sample_file (required): Sample file matching the new order.
- --output_folder: Output directory for new plots.
- Plot customization options (figure size, group order, etc.).

Outputs:

- Updated plots (heatmaps, dot plots) in the specified output folder, reflecting the new order or grouping.
- No new analysis is performed; only visualization is updated.

## Input format details:

CRISPRessoSea requires two main input files: a **target file** and a **sample (experiment) file**. Both can be provided as tab-delimited .txt or .xlsx files.

### Target File

**Required columns:**

| **Column** | **Description** |
| --- | --- |
| Guide | Name of the on-target guide (used to group on- and off-targets) |
| Sequence | Target DNA sequence (without PAM, either on-target or off-target) |
| PAM | Protospacer Adjacent Motif sequence |
| #MM | Number of mismatches between the on-target and this target (0 for on-target) |
| Locus | Genomic location in the format [chr:pos](vscode-file://vscode-app/c:/Users/u0351481/AppData/Local/Programs/Microsoft%20VS%20Code/resources/app/out/vs/code/electron-browser/workbench/workbench.html), [chr:+pos](vscode-file://vscode-app/c:/Users/u0351481/AppData/Local/Programs/Microsoft%20VS%20Code/resources/app/out/vs/code/electron-browser/workbench/workbench.html), or [chr:-pos](vscode-file://vscode-app/c:/Users/u0351481/AppData/Local/Programs/Microsoft%20VS%20Code/resources/app/out/vs/code/electron-browser/workbench/workbench.html) for strand information |

**Optional columns:**

| **Column** | **Description** |
| --- | --- |
| Target | Custom name for the target (used in plots/reports) |
| Mismatch_info | Human-readable description of mismatches and bulges (for reference only - not used in computation) |

**Notes:**

- Column names are case-insensitive.
- All required columns must be present.
- Additional columns will be ignored.
- - Locus strand information is not required or used by the pipeline. The strand-containing format is accepted because it is produced by some upstream tools.
- - The Locus information is used to assign targets to amplicon sequencing sites. This functionality can be disabled using the --allow_target_match_to_other_region_loc flag.

### Sample File

**Required columns:**

| **Column** | **Description** |
| --- | --- |
| Name | Unique name for the sample |
| fastq_r1 | Path to the R1 FASTQ file for this sample |

**Optional columns:**

| **Column** | **Description** |
| --- | --- |
| fastq_r2 | Path to the R2 FASTQ file (for paired-end reads) |
| group | Group label for the sample (used for grouping in plots/stats) |

**Notes:**

- The Name column must be unique for each sample.
- The fastq_r2 column is optional and only needed for paired-end data.
- The group column is optional but recommended for group-based analysis and plotting.
- Additional columns will be ignored.

# Supplementary Note 2: CRISPRessoSea comparison with CRISPRessoPooled^3^

CRISPRessoPooled can be run to analyze each sample separately. However, it is challenging to compare across CRISPRessoPooled samples, and labor-intensive copying of Excel files or custom scripting is required to aggregate these files.

Control

Treated

Supplemental Figure 1: CRISPRessoPooled plot output showing the number of reads and editing frequencies for a control (Fs2_ct1, left) and treated (Fs2_ep1, right) sample from the dataset in Figure 1.

As compared to CRISPRessoPooled, CRISPRessoSea streamlines analysis and interpretation of pooled experiments, requiring only one command to analyze and aggregate all data. This ensures that all samples are processed with comparable parameters, and eliminates the need for custom processing scripts that are hard to maintain. CRISPRessoSea also adds important plots showing the exact guide sequence changes at each site, and statistical measures for interpretation of editing frequencies, both features that are not present in CRISPRessoPooled.

# Supplementary Note 3: Figure generation details

Figure 2 was generated with the following CRISPRessoSea command:

CRISPRessoSea Process -t guides.txt -s Fs2_withgroup.txt -x ~/genomes/Homo_sapiens/UCSC/hg38/Sequence/Bowtie2Index/genome.fa -p 50 --plot_group_order ku,ep,ctl --plot_only_complete_targets -o final --crispresso_ignore_substitutions --crispresso_quantification_window_size 2

The following guide file was used:

Guide   Target  Sequence    PAM #MM Locus   Mismatch_info   Casoffinder_anno

Fs2 gRNA_Fs2    GCTGCAGAAGGGATTCCATG    AGG 0   chr11:+22625791 Mismatches: 0   FancS2_OB0_chr11_22625791

Fs2 gRNA_Fs2_OFF0   GCTGCAGAAGGGATTCCAaG    GGG 1   chr22:-36556947 Mismatches: 1   FancS2_OB1_chr22_36556947

Fs2 gRNA_Fs2_OFF1   GCTGCAaAAaGGATTCCAgG    GGG 3   chr3:+15170043  Mismatches: 3   FancS2_OB3_chr3_15170043

Fs2 gRNA_Fs2_OFF2   GacGCAGAAGGGAcTCCATG    GGG 3   chr6:-27764842  Mismatches: 3   FancS2_OB3_chr6_27764842

Fs2 gRNA_Fs2_OFF3   GCTGgAGgAaGGATTCCATG    GGG 3   chr11:+43924853 Mismatches: 3   FancS2_OB3_chr11_43924853

Fs2 gRNA_Fs2_OFF4   GgTaCAGAAGGGcTTCCATG    AGG 3   chr17:-59421384 Mismatches: 3   FancS2_OB3_chr17_59421384

Fs2 gRNA_Fs2_OFF5   tgTGaAGAAGGGtTTCCATG    AGG 4   chr11:-66842231 Mismatches: 4   FancS2_OB4_chr11_66842231

Fs2 gRNA_Fs2_OFF6   aCTGCAaAAtGGATTCCATG    GGG 3   chr1:+73763012  Mismatches: 3   FancS2_OB3_chr1_73763012

Fs2 gRNA_Fs2_OFF7   aCTGCtGAgaGGATTCCATG    AGG 4   chr1:+219319407 Mismatches: 4   FancS2_OB4_chr1_219319407

Fs2 gRNA_Fs2_OFF8   GCTGCtGAAcaGATTCCATG    GGG 3   chr3:+65860679  Mismatches: 3   FancS2_OB3_chr3_65860679

Fs2 gRNA_Fs2_OFF9   GCTGaAGAAGGaATTtCATG    GGG 3   chr6:-134027323 Mismatches: 3   FancS2_OB3_chr6_134027323

Fs2 gRNA_Fs2_OFF10  GtTGCAGcAaGaATTCCATG    AGG 4   chr6:+136470258 Mismatches: 4   FancS2_OB4_chr6_136470258

Fs2 gRNA_Fs2_OFF11  caaGCAGAAGGGATTCCAca    AGG 5   chr8:+124208462 Mismatches: 5   FancS2_OB5_chr8_124208462

Fs2 gRNA_Fs2_OFF12  GCcaCAGAAGGGATTCtATG    AAG 3   chr20:-45328648 Mismatches: 3   FancS2_OB3_chr20_45328648

The following sample file was used:

Name    R1  R2  group

Fs2_ct1 data/Fs2_ct1_R1.fastq.gz    data/Fs2_ct1_R2.fastq.gz    ctl

Fs2_ct2 data/Fs2_ct2_R1.fastq.gz    data/Fs2_ct2_R2.fastq.gz    ctl

Fs2_ct3 data/Fs2_ct3_R1.fastq.gz    data/Fs2_ct3_R2.fastq.gz    ctl

Fs2_ep1 data/Fs2_ep1_R1.fastq.gz    data/Fs2_ep1_R2.fastq.gz    ep

Fs2_ep2 data/Fs2_ep2_R1.fastq.gz    data/Fs2_ep2_R2.fastq.gz    ep

Fs2_ep3 data/Fs2_ep3_R1.fastq.gz    data/Fs2_ep3_R2.fastq.gz    ep

Fs2_ku1 data/Fs2_ku1_R1.fastq.gz    data/Fs2_ku1_R2.fastq.gz    ku

Fs2_ku2 data/Fs2_ku2_R1.fastq.gz    data/Fs2_ku2_R2.fastq.gz    ku

Fs2_ku3 data/Fs2_ku3_R1.fastq.gz    data/Fs2_ku3_R2.fastq.gz    ku

Figure 3 was generated with the following CRISPRessoSea command:

CRISPRessoSea Process -t guides.txt -s samples.txt -x ~/genomes/Homo_sapiens/UCSC/hg38/Sequence/Bowtie2Index/genome.fa -p 80 --crispresso_quantification_window_center -15 --crispresso_quantification_window_size 10

The following guide file was used:

Guide   Target  Sequence    PAM #MM Locus

AHR 1_AHR_ON    CTTACCATCAAAGAAGCTCT    TGG     chr7:-17310108

AHR 2_AHR_OB3   ATTACCA-CAAAAAAGCTCT    TAG 3.0 chr16:-75933020

AHR 3_AHR_OB1   CTT-CCATCAAAGAAGCTCT    TAG 1.0 chr11:+70210471

AHR 4_AHR_OB2   ATTACCA-CAAAGAAGCTCT    GAG 2.0 chr2:-154195669

AHR 5_AHR_OB3   CTTAGCAGCAAGGAAGCTCT    GAG 3.0 chr5:-160396396

AHR 6_AHR_OB2   CTTTCC-TCAAAGAAGCTCT    TAG 2.0 chr12:+100539440

AHR 7_AHR_OB3   GTTTCCACTCAAAGAAGCTCT   GGG 3.0 chr10:-128824262

AHR 8_AHR_OB4   CATTCCTGCAAAGAAGCTCT    CGG 4.0 chr11:+79834169

AHR 9_AHR_OB3   TTTTCCATCAGAGAAGCTCT    TAG 3.0 chr6:+45661665

AHR 10_AHR_OB3  CTTATC--CAAAGAAGCTCT    TGG 3.0 chr17:-72296462

CISH    11_CISH_ON  CTCACCAGATTCCCGAAGGT    AGG     chr3:+50608367

CISH    12_CISH_OB4 ATCACCTGATCCCCAAAGGT    GGG 4.0 chr11:-115143413

CISH    13_CISH_OB3 ATCATCAGATTCCCAAAGGT    TGG 3.0 chrX:+58004438

CISH    14_CISH_OB4 CACCCCAGGTTCCCAAAGGT    GGG 4.0 chr8:+38950841

CISH    15_CISH_OB4 CTCAACACGTTCCCAAAGGT    GAG 4.0 chr17:-32589308

CISH    16_CISH_OB4 CCCTCCAGATTCACAAAGGT    TGG 4.0 chr5:+174611504

CISH    17_CISH_OB4 TTAACCAGCTTCCCAAAGGT    TAG 4.0 chr8:+93883303

CISH    18_CISH_OB3 GTCACCAGATTCCCAAAAGT    CAG 3.0 chr2:+209849256

CISH    19_CISH_OB3 CTGACTCAGATTCCCGAAGGA   AGG 3.0 chr20:-63972489

CISH    20_CISH_OB4 CTCTCCACATTCCCAAAAGT    TAG 4.0 chr8:+63972762

CISH    21_CISH_OB3 CTCAGCAGTTCCCCGAAGGT    CGG 3.0 chr8:+1370273

KLRG1   22_KLRG1_ON CCTTACCTTGAGAAGTTTAG    AGG     chr12:-9009061

KLRG1   23_KLRG1_OB4    CCTGACCCTGAGAAATTTTG    AGG 4.0 chr10:+86310151

KLRG1   24_KLRG1_OB3    CCATTCCCTGAGAAGTTTAG    GAG 3.0 chrX:+98420426

KLRG1   25_KLRG1_OB2    CCTGACCTTGAGAAGTTTTG    GAG 2.0 chr11:+8604683

KLRG1   26_KLRG1_OB4    ACTAACTCTGAGAAGTTTAG    AAG 4.0 chr1:-72056449

KLRG1   27_KLRG1_OB4    ACGTACTGTGAGAAGTTTAG    CAG 4.0 chr17:-18921688

KLRG1   28_KLRG1_OB3    CCTGGCCTTGGGAAGTTTAG    CAG 3.0 chr7:+116838944

KLRG1   29_KLRG1_OB3    GCTGACCTTCAGAAGTTTAG    AGG 3.0 chr3:-111614554

KLRG1   30_KLRG1_OB3    CCTGGCCTT-AGAAGTTTAG    CAG 3.0 chr9:-73976414

KLRG1   31_KLRG1_OB4    CTTTGCTTTGAGAAGTTTAA    CAG 4.0 chr15:+81885031

KLRG1   32_KLRG1_OB4    ACCTACCTTGAGAAATTTTG    GGG 4.0 chr4:-11249199

TIGIT   33_TIGIT_ON CAGGCCTTACCTGAGGCGAG    GGG     chr3:-114294112

TIGIT   34_TIGIT_OB3    CAGGGCT-ACCTGAGGCTAG    AAG 3.0 chr16:-48165626

TIGIT   35_TIGIT_OB2    CCGG-CTTACCTGAGGCGAG    AAG 2.0 chr19:+46722063

TIGIT   36_TIGIT_OB4    CAAGGCTGACCTGAAGCGAG    AGG 4.0 chr17:+72123600

TIGIT   37_TIGIT_OB3    CAGACC--ACCTGAGGCGAG    GAG 3.0 chr8:-772548

TIGIT   38_TIGIT_OB4    CAGGGCAGACCTGGGGCGAG    GAG 4.0 chr12:+108807817

TIGIT   39_TIGIT_OB4    CGGGACTGACCTGAGGTGAG    AGG 4.0 chr2:+222220129

TIGIT   40_TIGIT_OB3    CAGGTCT--CCTGAGGCGAG    GGG 3.0 chr11:-46696503

TIGIT   41_TIGIT_OB4    CGGGACCTCCCTGAGGCGAG    TGG 4.0 chr6:-44561968

TIGIT   42_TIGIT_OB3    CTGG-CTTACCTGAGGTGAG    TAG 3.0 chr2:+135757726

TIGIT   43_TIGIT_OB2    CTGGCCTTACCTGAGGAGAG    GAG 2.0 chr2:-46347053

PD1 44_PD1_ON   CACCTACCTAAGAACCATCC    TGG     chr2:+241858755

PD1 45_PD1_OB4  CAGCAATCTCAGAACCATCC    CAG 4.0 chrX:-19289554

PD1 46_PD1_OB1  CACCTGACCTAAGAACCATCC   AGG 1.0 chr15:-92446127

PD1 47_PD1_OB3  CACCCA--TAAGAACCATCC    TGG 3.0 chrX:-6802047

PD1 48_PD1_OB3  CATCAACCTCAGAACCATCC    CAG 3.0 chr4:+79274438

PD1 49_PD1_OB4  CCCCTACGCAACAACCATCC    AGG 4.0 chr3:+87089026

PD1 50_PD1_OB3  CACGCACCTCAGAACCATCC    CAG 3.0 chr1:+24158138

PD1 51_PD1_OB4  CCTCTACACAAGAACCATCC    TAG 4.0 chr13:+83470250

PD1 52_PD1_OB3  AACCTCCCTCAAGAACCATCC   AAG 3.0 chr18:-45509718

PD1 53_PD1_OB3  CAGGTACCTAA-AACCATCC    CAG 3.0 chr1:+230732931

PD1 54_PD1_OB4  CACTCACTTAAGTACCATCC    TGG 4.0 chr3:-148945388

The following sample file was used:

217-0_S22   data/217-0_S22_L001_R1_001.fastq.gz data/217-0_S22_L001_R2_001.fastq.gz

217-6_S28   data/217-6_S28_L001_R1_001.fastq.gz data/217-6_S28_L001_R2_001.fastq.gz

4_0_S1  data/4_0_S1_L001_R1_001.fastq.gz    data/4_0_S1_L001_R2_001.fastq.gz

4_6_S7  data/4_6_S7_L001_R1_001.fastq.gz    data/4_6_S7_L001_R2_001.fastq.gz

6_0_S8  data/6_0_S8_L001_R1_001.fastq.gz    data/6_0_S8_L001_R2_001.fastq.gz

6_6_S14 data/6_6_S14_L001_R1_001.fastq.gz   data/6_6_S14_L001_R2_001.fastq.gz

8_0_S15 data/8_0_S15_L001_R1_001.fastq.gz   data/8_0_S15_L001_R2_001.fastq.gz

8_6_S21 data/8_6_S21_L001_R1_001.fastq.gz   data/8_6_S21_L001_R2_001.fastq.gz

# Supplementary Note 4: Simulation study

Synthetic amplicon sequencing data were generated using a custom python script (01_make_simulations.py) with a fixed random seed to ensure reproducibility. Ten independent reference amplicons (100 bp each) were created by random nucleotide sampling. For each amplicon, a guide sequence was defined at positions 33–53 (0-based coordinates), spanning the intended editing site, with simulated edits occurring at positions 50–51.

For each target, sequencing reads were simulated at a depth of 10,000 reads per sample. Editing rates were modeled across a dilution series (100%, 50%, 25%, 10%, 5%, 1%, 0.1%, 0.01%, 0.001%, and 0.0001%), alongside background mutation rates of 0%, 1%, 5%, and 10%. Five biological replicates were generated per background condition, each consisting of an “edited” sample and a matched “unedited” control.

At the read level, editing and background mutation events were independently assigned according to their respective probabilities. Reads selected for mutation were modified by introducing deletions centered at one of the two editing positions (chosen at random), with deletion half-lengths of 1–3 bp sampled uniformly. Reads without mutations retained the reference sequence, and all reads were assigned uniformly high base quality scores.

Simulated datasets were analyzed using CRISPRessoSea to evaluate the performance of the statistical methods described above. Code and full simulation details are available at: <https://github.com/clementlab/CRISPRessoSea/tree/main/simulation>

Following simulation, we evaluated statistical approaches implemented in CRISPRessoSea for detecting editing across a range of true editing rates and background noise levels. Performance is summarized as heatmaps showing detection behavior across these parameters, including replicate consistency and statistical significance where applicable.

**Fig S4.1a: Performance of a hard cutoff (≥0.01%) across simulated editing and noise levels (n = 3 replicates).** Heatmaps show the number of replicates (out of 3) in which editing is detected above a 0.01% threshold. The x-axis indicates the true editing rate (1e-6 (0.001% editing) to 1.0 (100% editing)), and the y-axis indicates the background noise rate (0 to 0.1 (10% noise)). **Left:** Edited samples. At very low true editing rates and minimal noise, editing is inconsistently detected (e.g., 0/3–2/3 replicates), whereas at higher editing rates or increased noise, all replicates are classified as edited (3/3). **Right:** Unedited control samples. In the absence of noise, no replicates are classified as edited (0/3). However, as noise exceeds ~0.01, all replicates are classified as edited (3/3), indicating that this threshold may not be appropriate for samples with high noise rates.

**Fig S4.1b:** **Performance of a hard cutoff (≥0.01%) across simulated editing and noise levels (n = 5 replicates).** Heatmaps as in S4.1a except for showing data for 5 replicates.

**Fig S4.2a: Performance of the mean-difference (mean_diff ≥ 0.01%) approach across simulated editing and noise levels (n = 3 replicates).**
Heatmaps (left) summarize the mean difference between observed and background signal, (middle) the fraction of replicates called significant, and (right) the number of replicates with insufficient data (NA). The x-axis indicates the true editing rate (1e−6 to 1.0), and the y-axis indicates the background noise rate (0 to 0.1).

The mean-difference increases with true editing rate and remains stable across noise levels, with near-zero values at very low editing. Correspondingly, significance is inconsistently detected only at the lowest editing rates but becomes consistent across replicates at ≥1e−3, while no NA values are observed under any condition.

**Fig S4.2b: Performance of the mean-difference (mean_diff ≥ 0.01%) approach across simulated editing and noise levels (n = 5 replicates).** Heatmaps show (left) mean p-values, (middle) fraction of replicates called significant, and (right) NA counts. The x-axis indicates the true editing rate (1e−6 to 1.0), and the y-axis indicates the background noise rate (0 to 0.1) as in S4.2a but with n = 5 replicates.

**Fig. S4.3a: Performance of the t-test (significance cutoff of p<0.05, BH corrected) across simulated editing and noise levels (n = 3 replicates).** Heatmaps show (left) mean p-values, (middle) fraction of replicates called significant, and (right) NA counts. The x-axis indicates the true editing rate (1e−6 to 1.0), and the y-axis indicates the background noise rate (0 to 0.1) as in Supp 4.2a. Mean p-values decrease sharply with increasing true editing rate, with high variability and generally non-significant values at low editing levels, particularly under higher noise. Correspondingly, significance is not detected at low editing rates but becomes consistent across replicates at ≥0.05. The NA p-values at the lowest editing rates with zero background noise happen because both the edited and unedited groups are all zeros in those cases. At 1e-5 and 1e-6 editing rates, the expected number of edited reads is so low that the simulated edited samples often contain no detectable edited reads at all, making them identical to the zero-noise controls.

**Fig. S4.3b | Performance of the t-test (significance cutoff of p<0.05, BH corrected) across simulated editing and noise levels (n = 5 replicates).** Heatmaps show (left) mean p-values, (middle) fraction of replicates called significant, and (right) NA counts. The x-axis indicates the true editing rate (1e−6 to 1.0), and the y-axis indicates the background noise rate (0 to 0.1) as in Supp 4.3a but with n = 5 replicates.

**Fig. S4.4a: Performance of the Mann-Whitney U test (significance cutoff of p<0.05, BH corrected) across simulated editing and noise levels (n = 3 replicates).** Heatmaps show (left) mean p-values, (middle) fraction of replicates called significant, and (right) NA counts. The x-axis indicates the true editing rate (1e−6 to 1.0), and the y-axis indicates the background noise rate (0 to 0.1) as in Supp 4.2a. Note that average p-values (left panel) are uncorrected. Mean p-values remain high and largely insensitive to increasing true editing rate, particularly at low-to-moderate editing levels, indicating limited power under these conditions. Consistently, no replicates are classified as significant across all tested parameters (0/1 throughout), while no NA values are observed, demonstrating stable but overly conservative behavior.

**Fig. S4.4b | Performance of the Mann-Whitney U test (significance cutoff of p<0.05, BH corrected) across simulated editing and noise levels (n = 5 replicates).** Heatmaps show (left) mean p-values, (middle) fraction of replicates called significant, and (right) NA counts. The x-axis indicates the true editing rate (1e−6 to 1.0), and the y-axis indicates the background noise rate (0 to 0.1) as in Supp 4.4a but with n = 5 replicates. Mean p-values decrease with increasing true editing rate, particularly at ≥0.01, indicating improved sensitivity compared to the n = 3 setting, though variability persists at low editing levels and higher noise. Correspondingly, significance is not detected at very low editing rates but becomes consistent across replicates at ≥0.05, while no NA values are observed across all tested conditions.

**Fig. S4.5a: Performance of the negative binomial test (significance cutoff of p<0.05, BH corrected) across simulated editing and noise levels (n = 3 replicates).** Heatmaps show (left) mean p-values, (middle) fraction of replicates called significant, and (right) NA counts. The x-axis indicates the true editing rate (1e−6 to 1.0), and the y-axis indicates the background noise rate (0 to 0.1) as in Supp 4.2a. Note that average p-values (left panel) are uncorrected. Mean p-values decrease progressively with increasing true editing rate, with a clear dependence on background noise such that higher noise shifts significance to higher editing thresholds. Accordingly, significance is only detected at sufficiently high editing rates relative to noise (diagonal boundary), indicating good specificity but reduced sensitivity under noisy conditions.

**Fig. S4.5b | Performance of the negative binomial test (significance cutoff of p<0.05, BH corrected) across simulated editing and noise levels (n = 5 replicates).** Heatmaps show (left) mean p-values, (middle) fraction of replicates called significant, and (right) NA counts. The x-axis indicates the true editing rate (1e−6 to 1.0), and the y-axis indicates the background noise rate (0 to 0.1) as in Supp 4.3a but with n = 5 replicates.

# References:

1. Bae S, Park J, Kim JS. Cas-OFFinder: a fast and versatile algorithm that searches for potential off-target sites of Cas9 RNA-guided endonucleases. Bioinformatics. 2014;30(10):1473–1475. PMCID: PMC4016707

2. Langmead B, Salzberg SL. Fast gapped-read alignment with Bowtie 2. Nat Methods. 2012;9(4):357–359. PMCID: PMC3322381

3. Clement K, Rees H, Canver MC, Gehrke JM, Farouni R, Hsu JY, Cole MA, Liu DR, Joung JK, Bauer DE, Pinello L. CRISPResso2 provides accurate and rapid genome editing sequence analysis. Nat Biotechnol. 2019;37(3):224–226. PMCID: PMC6533916
